# Protein model accuracy estimation empowered by deep learning and inter-residue distance prediction in CASP14

**DOI:** 10.1038/s41598-021-90303-6

**Published:** 2021-05-25

**Authors:** Xiao Chen, Jian Liu, Zhiye Guo, Tianqi Wu, Jie Hou, Jianlin Cheng

**Affiliations:** 1 Department of Electrical Engineering and Computer Science, University of Missouri-Columbia, Columbia, 65201 USA; 2 Department of Computer Science, Saint Louis University, Saint. Louis, MO 63103 USA

**Keywords:** Bioinformatics, Protein folding, Protein structure predictions

## Abstract

The inter-residue contact prediction and deep learning showed the promise to improve the estimation of protein model accuracy (EMA) in the 13th Critical Assessment of Protein Structure Prediction (CASP13). To further leverage the improved inter-residue distance predictions to enhance EMA, during the 2020 CASP14 experiment, we integrated several new inter-residue distance features with the existing model quality assessment features in several deep learning methods to predict the quality of protein structural models. According to the evaluation of performance in selecting the best model from the models of CASP14 targets, our three multi-model predictors of estimating model accuracy (MULTICOM-CONSTRUCT, MULTICOM-AI, and MULTICOM-CLUSTER) achieve the averaged loss of 0.073, 0.079, and 0.081, respectively, in terms of the global distance test score (GDT-TS). The three methods are ranked first, second, and third out of all 68 CASP14 predictors. MULTICOM-DEEP, the single-model predictor of estimating model accuracy (EMA), is ranked within top 10 among all the single-model EMA methods according to GDT-TS score loss. The results demonstrate that inter-residue distance features are valuable inputs for deep learning to predict the quality of protein structural models. However, larger training datasets and better ways of leveraging inter-residue distance information are needed to fully explore its potentials.

## Introduction

In a protein structure prediction process, the estimation of model accuracy (EMA) or model quality assessment (QA) without the knowledge of native/true structures is important for selecting good tertiary structure models from many predicted models. EMA also provides valuable information for researchers to apply protein structural models in biomedical research. The previous studies have shown that the accurate estimation of the quality of a pool of predicted protein models is challenging^1,2^. The performance of EMA methods largely depends on two major factors: the quality of predicted structures in a model pool and the precision of the methods for model ranking^2,3^. The EMA methods had demonstrated the effectiveness in picking the high-quality models when the predicted models are more accurate. EMA methods can more readily distinguish the good-quality models from incorrectly folded structures using various existing model quality features identified from the models, including stereo-chemical correctness, the atomic statistical potential at the main chain and side chain^4–10^, atomic solvent accessibility, secondary structure agreement, and residue-residue contacts^11^. Conversely, these structural features become more conflicting on those poorly predicted models, which are commonly observed in a model pool consisting of predominantly low-quality models. Combining multiple individual model quality features has been demonstrated as an effective technique to provide a more robust and accurate estimation of model quality^11–15^. In recent years, the noticeable improvement has been achieved due to the feature integration by deep learning and the advent of the accurate prediction of inter-residue geometry constraints.

In the 13th Critical Assessment of Protein Structure Prediction (CASP13), the inter-residue contact information and deep learning were the key for DeepRank^17^ to achieve the best performance in ranking protein structural models with the minimum loss of GDT-TS score^18^. Recently, inter-residue distance predictions have been used with more deep learning methods for the estimation of model accuracy^19–21^. For instance, ResNetQA^19^ applied the combination of 2D and 1D deep residual networks to predict the local and global protein quality score simultaneously. It was trained on the data from three sources: CASP, CAMEO^48^, and CATH^49^. In the CASP14 experiment, DeepAccNet^20^, a deep residual network to predict the local quality score, achieved the best performance in terms of Local Distance Difference Test (LDDT)^47^ score loss.

To investigate how residue-residue distance/contact features may improve protein model quality assessment with deep learning, we developed several EMA predictors to evaluate different ways of using contact and distance predictions as features in the 2020 CASP14 experiment. Some of these predictors are based on the features used in our CASP13 EMA predictors, while others use the new contact/distance-based features^22^ or new image similarity-derived features^23–26^ by treating predicted inter-residue distance maps as images and calculating the similarity between the distance maps predicted from protein sequences and the distance maps directly computed from the 3D coordinates of a model, which have not been used before in the field. All the methods predict a normalized GDT-TS score for a model of a target using deep learning, which estimates the quality of the model in the range from 0 (worst) to 1 (best).

According to the nomenclature in the field, these CASP14 MULTICOM EMA predictors can be classified into two categories: multi-model methods (MULTICOM-CLUSTER, MULTICOM-CONSTRUCT, MULTICOM-AI, MULTICOM-HYBRID) that use some features based on the comparison between multiple models of the same protein target as input and single-model methods (MULTICOM-DEEP and MULTICOM-DIST) that only use the features derived from a single model without referring to any other model of the target. Multi-model methods had performed better than single-model methods in most cases in the past CASP experiments^17^. However, multi-model methods may perform poorly when there are only a few good models in the model pool of a target, while the prediction of single-model methods for a model is not affected by other models in the pool. Moreover, single-model methods can predict the absolute quality score for a single protein model^27,28^, while the score predicted by multi-model methods for a model depends on other models in the model pool. In the following sections, we describe the technical details of these two kinds of methods, analyze their performance in the CASP14 experiment, and report our findings.

## Methods

### The pipeline and features for estimation of model accuracy

**Figure 1:**
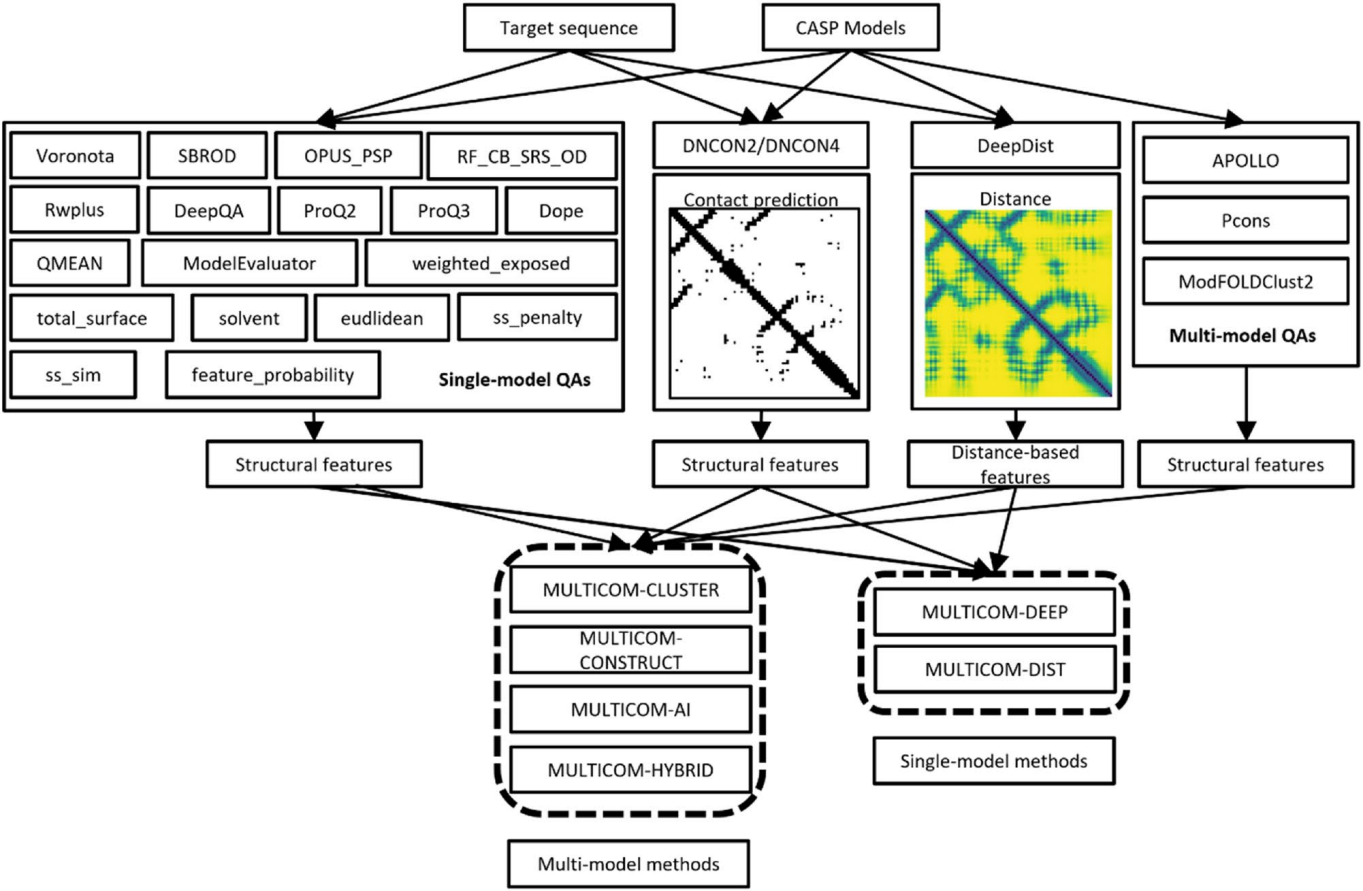
The pipeline of MULTICOM EMA predictors. Multi-model methods (MULTICOM-CLUSTER/CONSTRUCT/AI/HYBRID) uses both single-model quality assessment features and multi-model quality assessment features, while single-model methods (MULTICOM-DEEP/DIST) only uses the single-model quality features.

Figure 1 shows the pipeline for MULTICOM EMA predictors. When a protein target sequence and a pool of predicted structural models for the target are received, a MULTICOM EMA predictor calls an inter-residue distance predictor—DeepDist^29^ and/or an inter-residue contact predictor—DNCON2^30^/DNCON4^31^ to predict the distance map and/or contact map for the target. Given the contact prediction, it first calculates the percentage of predicted inter-residue contacts (i.e., short-range, medium-range, and long-range contacts) occurring in the structural model as in our early work^17^. Furthermore, it applies several novel metrics of describing the similarities or difference between the predicted distance map (PDM) and a structural model’s distance map (MDM) as features, such as the Pearson’s correlation between PDM and MDM, the image-based similarity between two distance maps including the distance-based DIST descriptor^23^, Oriented FAST and Rotated BRIEF (ORB)^24^, and PHASH^25^, and PSNR SSIM^26^ as well as root mean square error (RMSE).

Other non-distance/contact features used in DeepRank are also generated for the predictors, which include single-model features, i.e., SBROD^10^, OPUS_PSP^8^, RF_CB_SRS_OD^9^, Rwplus^6^, DeepQA^32^, ProQ2^33^, ProQ3^34^, Dope^5^, Voronota^35^, ModelEvaluator^27^, QMEAN^36^, solvent accessibility score (i.e., solvent) generated by SSpro4^37^ and DSSP^38^, regular secondary structure (helix and beta sheet) penalty score (i.e., ss_penalty), secondary structure similarity score (i.e., ss_sim)^39^, paired Euclidean distance score (i.e., euclidean), total surface score (i.e., total_surface), weighted exposed surface area score (i.e., weighted_exposed), and an average feature probability density score (i.e., feature_probability)^16^. The three multi-model features are APOLLO^39^, Pcons^40^, and ModFOLDClust2^41^. Different combinations of the features described above are used with deep learning to predict the GDT-TS score of a model, resulting in multiple MULTICOM EMA predictors. Table 1 is the summary of MULTICOM EMA predictors’ features information and data information. Table 2 shows each model’s features’ details.

**Table 1:** The number of features and training/test data used by MULTICOM EMA predictors.

Method	Number of features	Training data	Test data
MULTICOM-CONSTRCUT	18	CASP8-11 (428 targets, 84,098 decoys)	CASP12 (62 targets, 9617 decoys)
MULTICOM-CLUSTER	21	CASP8-11 (428 targets, 84,098 decoys)	CASP12 (62 targets, 9617 decoys)
MULTICOM-AI	19	CASP8-12 (490 targets, 93,715 decoys)	CASP13 (80 targets, 11,750 decoys)
MULTCOM-HYBRID	31	CASP8-12 (490 targets, 93,715 decoys)	CASP13 (80 targets, 11,750 decoys)
MULTICOM-DEEP	29	CASP8-12 (490 targets, 93,715 decoys)	CASP13 (80 targets, 11,750 decoys)
MULTICOM-DIST	17	CASP8-12 (490 targets, 93,715 decoys)	CASP13 (80 targets, 11,750 decoys)

The details of the features can be found in Table 2. The structural models of CASP8-13 were used in training and test. MULTCIOM-AI used 20% of training targets as validation dataset, while the other five predictors used 10% of training targets as validation dataset.

**Table 2:** Features used by six MULTICOM EMA predictors.

Feature	Category	MULTICOM-CLUSTER	MULTICOM-CONSTRUCT	MULTICOM -AI	MULTICOM-HYBRID	MULTICOM-DEEP	MULTICOM-DIST
dist_gist	Distance-based single model feature	**×**	**×**	**×**	**✓**	**✓**	**✓**
dist_precl2_long		**×**	**×**	**×**	**✓**	**✓**	**✓**
dist_precl2		**×**	**×**	**×**	**✓**	**✓**	**✓**
dist_ssim		**×**	**×**	**×**	**✓**	**✓**	**✓**
dist_psnr		**×**	**×**	**×**	**✓**	**✓**	**✓**
dist_recall_long		**×**	**×**	**×**	**✓**	**✓**	**✓**
dist_orb_num		**×**	**×**	**×**	**✓**	**✓**	**✓**
dist_pearson		**×**	**×**	**×**	**✓**	**✓**	**✓**
dist_phash		**×**	**×**	**×**	**✓**	**✓**	**✓**
dist_recall		**×**	**×**	**×**	**✓**	**✓**	**✓**
dist_rmse		**×**	**×**	**×**	**✓**	**✓**	**✓**
Correlation_feature		**×**	**×**	**✓**	**×**	**×**	**×**
contact_short-range	Contact-based single model feature	**✓ #**	**✓ #**	**✓ $**	**✓ $**	**✓ $**	**×**
contact_medium-range		**✓ #**	**✓ #**	**✓ $**	**✓ $**	**✓ $**	**×**
contact_long-range		**✓ #**	**✓ #**	**✓ $**	**✓ $**	**✓ $**	**×**
Voronota	Other single-model feature	**✓**	**✓**	**✓**	**✓**	**✓**	**✓**
SBROD		**✓**	**✓**	**✓**	**✓**	**✓**	**✓**
OPUS_PSP		**✓**	**✓**	**✓**	**✓**	**✓**	**✓**
RF_CB_SRS_OD		**✓**	**✓**	**✓**	**✓**	**✓**	**✓**
Rwplus		**✓**	**✓**	**✓**	**✓**	**✓**	**✓**
DeepQA		**✓**	**×**	**✓**	**✓**	**✓**	**×**
ProQ2		**✓**	**×**	**×**	**✓**	**✓**	**×**
ProQ3		**✓**	**×**	**×**	**✓**	**✓**	**×**
Dope		**✓**	**×**	**✓**	**✓**	**✓**	**✓**
QMEAN		**×**	**✓**	**×**	**×**	**×**	**×**
ModelEvaluator		**×**	**✓**	**×**	**×**	**×**	**×**
weighted_exposed		**✓**	**✓**	**✓**	**✓**	**✓**	**×**
total_surface		**✓**	**✓**	**✓**	**✓**	**✓**	**×**
solvent		**✓**	**✓**	**✓**	**✓**	**✓**	**×**
euclidean		**✓**	**✓**	**✓**	**✓**	**✓**	**×**
ss_penalty		**✓**	**✓**	**✓**	**✓**	**✓**	**×**
ss_sim		**✓**	**✓**	**✓**	**✓**	**✓**	**×**
feature_probability		**×**	**✓**	**×**	**×**	**×**	**×**
APOLLO	Multi-model feature	**✓**	**×**	**×**	**✓**	**×**	**×**
Pcons		**✓**	**✓**	**✓**	**✓**	**×**	**×**
ModFOLDClust2		**✓**	**×**	**✓**	**✓**	**×**	**×**

The features are divided into four categories: distance-based single-model features, contact-based single-model features, other single-model features, and multi-model features. **✓**: a feature used by a predictor. **×**: a feature not used by a predictor. #: features based on contacts predicted by DNCON2. $: features based on contacts predicted by DNCON4.

**Figure 2:**
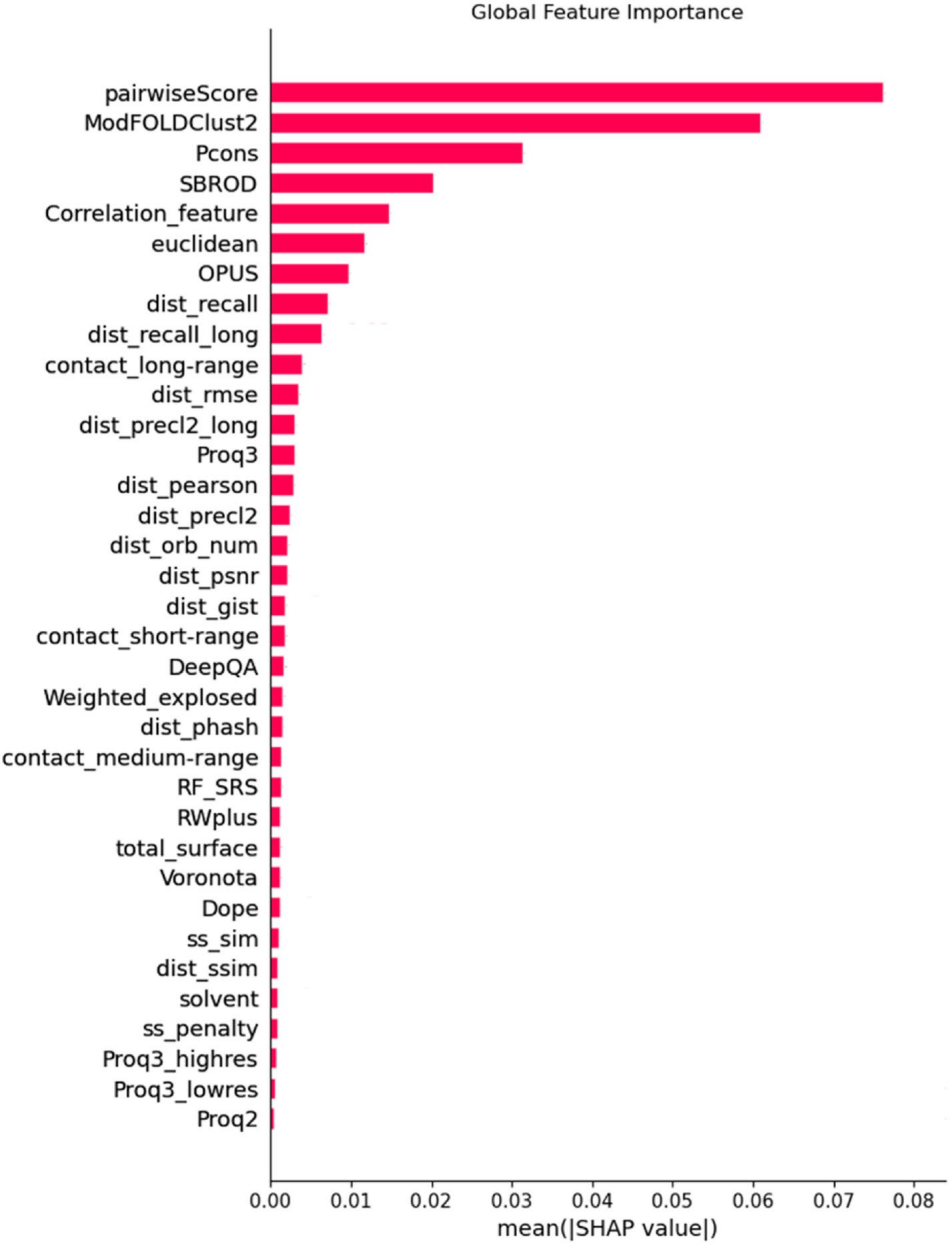
A bar plot of the average SHAP values of the features.

The importance of the features is assessed by SHAP value^45^. SHAP value represents a feature’s contribution to an EMA model’s output. A higher SHAP value means the feature has a higher impact on the prediction result. Figure 2 shows all features’ SHAP values, which were calculated by TreeExplainer^45^ and LightGBM^46^ on CASP8-13 models. Several features derived from the protein distance maps (Correlation_feature, dist_recall, dist_recall_long, contact_long-range) are ranked 5th, 8th, 9th and 10th about all the 36 features. 8 of the rest distance/contact-based features are ranked in top 20.

In order to handle a partial structural model that contains only the coordinates for a portion of the residues of a target, the score for a partial model predicted by a deep learning predictor is normalized by multiplying it by a ratio equal to the number of residues in the partial model divided by the total number of the residue of the target (i.e., target sequence length). For very large protein targets (i.e., T1061, T1080, T1091), we adopted the domain-based analysis to evaluate the quality of their models because it was impossible to evaluate their full-length models within the limited time window of CASP14. The MULTICOM predictors divide a full-length model into domains according to the domain boundaries predicted from the full-length sequence and evaluates their quality separately. The average score of the domains is considered as the final quality score of the full-length model.

### Deep learning training and prediction

The structural models of protein targets of CASP8-CASP11/CASP12 were used as a training dataset to train and validate the deep learning EMA methods (Table 1). We also evaluated all predictors on the CASP13 dataset before they were blindly tested in CASP14.

The training dataset was split into K equal-size folds. Each fold was used as the validation set for parameter tuning while the remaining K-1 folds were used to train a deep learning model to predict the quality score. This process was repeated K times, yielding K-trained EMA predictors each validated on one-fold (i.e., K-fold cross-validation, MULTICOM-AI applies 5-fold cross-validation, rest five models use 10-folds cross-validation) Both MULTICOM-AI and MULTICOM-CLUSTER use the ensemble approach to average the outputs of K predictors to predict the model quality.

Moreover, the remaining EMA predictors (MULTICOM-CONSTRUCT, MULTICOM-DEEP, MULTICOM-DIST, MULTICOMHYBRID) used a two-stage training strategy, adding another round of training (stage-2 training) on top of the modeling trained above (i.e., stage-1 training). The outputs of K predictors in stage-1 are combined with the original features to be used as input for another deep learning model in stage-2 to predict final quality score. All the deep learning models in stage-2 were trained on the same structural models as in stage-1.

### Implementation of MULTICOM EMA predictors

MULTICOM-CONSTRUCT and MULTICOM-CLUSTER use the same deep learning architecture as in DeepRank^17^. They were trained using the 10-fold cross-validation. However, MULTICOM-CONSTRUCT uses the average output of the 10 predictors trained in the stage-1 training as prediction, but MULTICOM-CLUSTER applies the deep learning model trained with the two-stage training strategy to make the prediction. MULTICOM-AI is built upon a variant of DeepRank^22^. Its deep network was trained with 5-fold cross-validation. In each round of training, fours folds data were treated as training dataset and rest one was validation set. MULTICOM-AI used five dense layers. To improve training, a batch normalization layer was inserted between second and third dense layer. Two drop-out layers were also added to reducing overfitting.

MULTICOM-HYBRID, MULTICOM-DEEP, and MULTICOM-DIST use some input features that are quite different from MULTICOM-CONSTRUCT, MULTICOM-CLUSTER and MULTICOM-AI (see Table 1). First, DNCON2 is replaced with its improved version, DNCON4, to make contact predictions for contact-based features. The inter-residue distance-based features (i.e., SSIM, PSNR, GIST, RMSE, Recall, Precision, PHASH, Pearson correlation, ORB) calculated from distance maps predicted by DeepDist are also used as their input features. MULTICOM-HYBRID uses the new contact-based features and the new distance-based features as well as the nine single-model quality scores and the three multi-model features of DeepRank to make predictions. Because it uses the multi-model features as input, it is a multi-model method. MULTICOM-DEEP uses the same features as MULTICOM-HYBRID except that the three multi-model features are removed. Therefore, MULTICOM-DEEP is a single-model method. MULTICOM-DIST is a light version of MULTICOM-DEEP. It uses a subset of the features of MULTICOM-DEEP, excluding several features including DeepQA, ProQ2, ProQ3, contact matching scores that take quite some time to generate. MULTICOM-HYBRID, MULTICOM-DEEP, MULTICOM-DIST were trained on CASP8-12 protein models and tested on CASP13 models, yielding the similar GDT-TS loss on the test dataset (i.e., 0.0487, 0.0533, 0.0503, respectively).

## Results

### Evaluation data and metrics

MULTICOM EMA predictors blindly participated in the CASP14 EMA prediction category from May to July 2020. CASP14 evaluated EMA methods in two stages^42^. In stage 1, 20 models of each target with very different quality were sent to the registered EMA predictors to predict their quality. In stage 2, top 150 models selected by a simple consensus EMA method for each target were used for the EMA predictors to predict their quality. Because CASP14 only released the official evaluation results on stage-2 models, we analyze all EMA methods on the stage-2 models of 69 valid targets in this study. A CASP14 target may have one single domain or multiple domains. The domains are classified into three categories: (1) template-based modeling (TBM) domains—the regular domains that have known structure templates in the Protein Data Bank (PDB)^43^ (TBM domains are further classified into TBM-easy and TBM-hard categories according to the difficulty of predicting their tertiary structures); (2) free modeling (FM) domains—the very hard domains that do not have any known structure templates in the PDB; and (3) something between the two (FM/TBM), which may have some very weak templates that cannot be recognized by existing template-identification methods. If a target contains multiple domains of different difficulty categories, it is classified into the most difficult category of its domains in this study.

We downloaded the official evaluation results of the CASP14 EMA predictors from the CASP14 website, analyzed MULTICOM EMA predictors’ performance, and compared them with other EMA predictors. We use the average loss of the

GDT-TS score of an EMA predictor over all the CASP14 targets as the main metric to evaluate its performance. The GDT-TS loss of a predictor on a target is the absolute difference between the true GDT-TS score of the No. 1 model selected from all the models of the target by the predicted GDT-TS scores and the true GDT-TS score of the best model of the target. A loss of 0 means the best model in the model pool of a target is chosen by an EMA predictor, which is the ideal situation. The average GDT-TS loss of a predictor on all the CAS14 targets is used to evaluate how well it can select or rank protein models. In addition to the GDT-TS loss, we also use the average Pearson’s correlation between the predicted GDT-TS scores of the models of a target and their true GDT-TS scores over the CASP14 targets to evaluate the EMA methods.

### GDT-TS loss and Pearson’s correlation of the MULTICOM EMA predictors in CASP14

**Figure 3:**
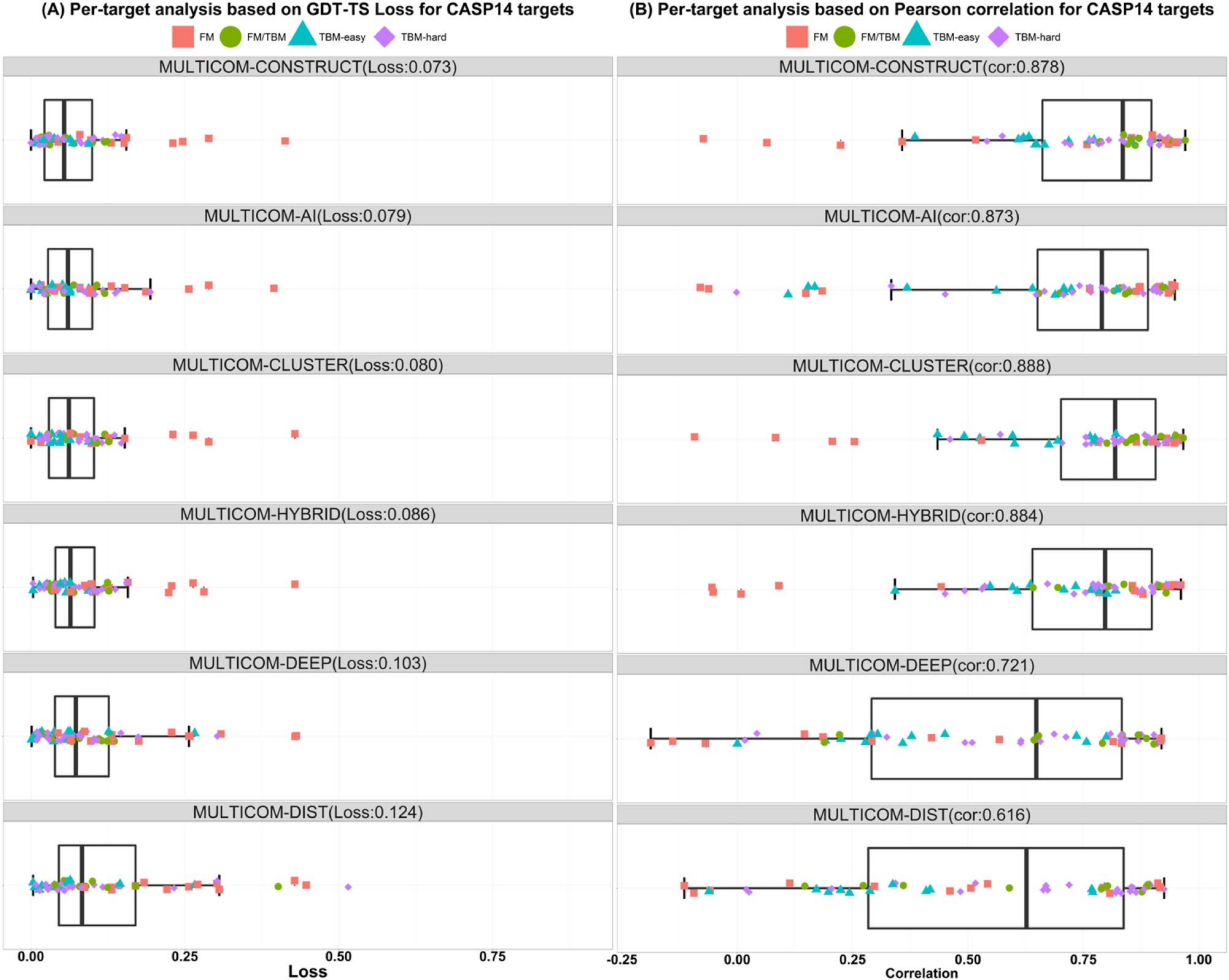
The boxplots of MULTICOM predictors’ performance on CASP14 targets. (**A**) GDT-TS score loss. (**B**) Pearson’s correlation score. Different colors/shapes denote different kinds of targets.

Boxplots in Fig. 3A show the GDT-TS loss of each target, average loss, and variation of the loss for the six MULTICOM

EMA predictors on all the CASP14 targets. MULTICOM-CONSTRUCT, AI, CLUSTER, HYBRID, DEEP and DIST attain 0.0734, 0.0792, 0.0806, 0.086, 0.104, and 0.124 (GDT-TS) loss on average, respectively. Overall, the four multi-model methods (MULTICOM-CONSTRUCT, AI, CLUSTER, HYBRID) perform better than the two single-model methods (MULTICOM-DEEP, DIST), among which MULTICOM-CONSTRUCT performed best in terms of the GDT-TS loss.

Figure 3B plots the Pearson’s correlation scores of the CASP14 targets for each MULTICOM predictor. MULTICOM-CLUSTER obtains the highest global correlation coefficient (0.888) and MULTICOM-DIST’s correlation coefficient (0.616) is the lowest. The four multi-model methods’ correlation scores are close (i.e., MULTICOM-CONSTRUCT: 0.878, MULTICOM-AI: 0.873, MULTICOM-HYBRID: 0.884, MULTICOM-CLUSTER: 0.888). Their high correlation scores indicate a strong positive correlation relationship between the true GDT-TS scores and predicted GDT-TS scores. The two single-model methods perform worse than the multi-model methods. MULTICOM-DEEP, MULTICOM-DIST achieve a correlation score of 0.721 and 0.611, respectively.

### Comparison between multi-model methods and single-model methods

**Figure 4:**
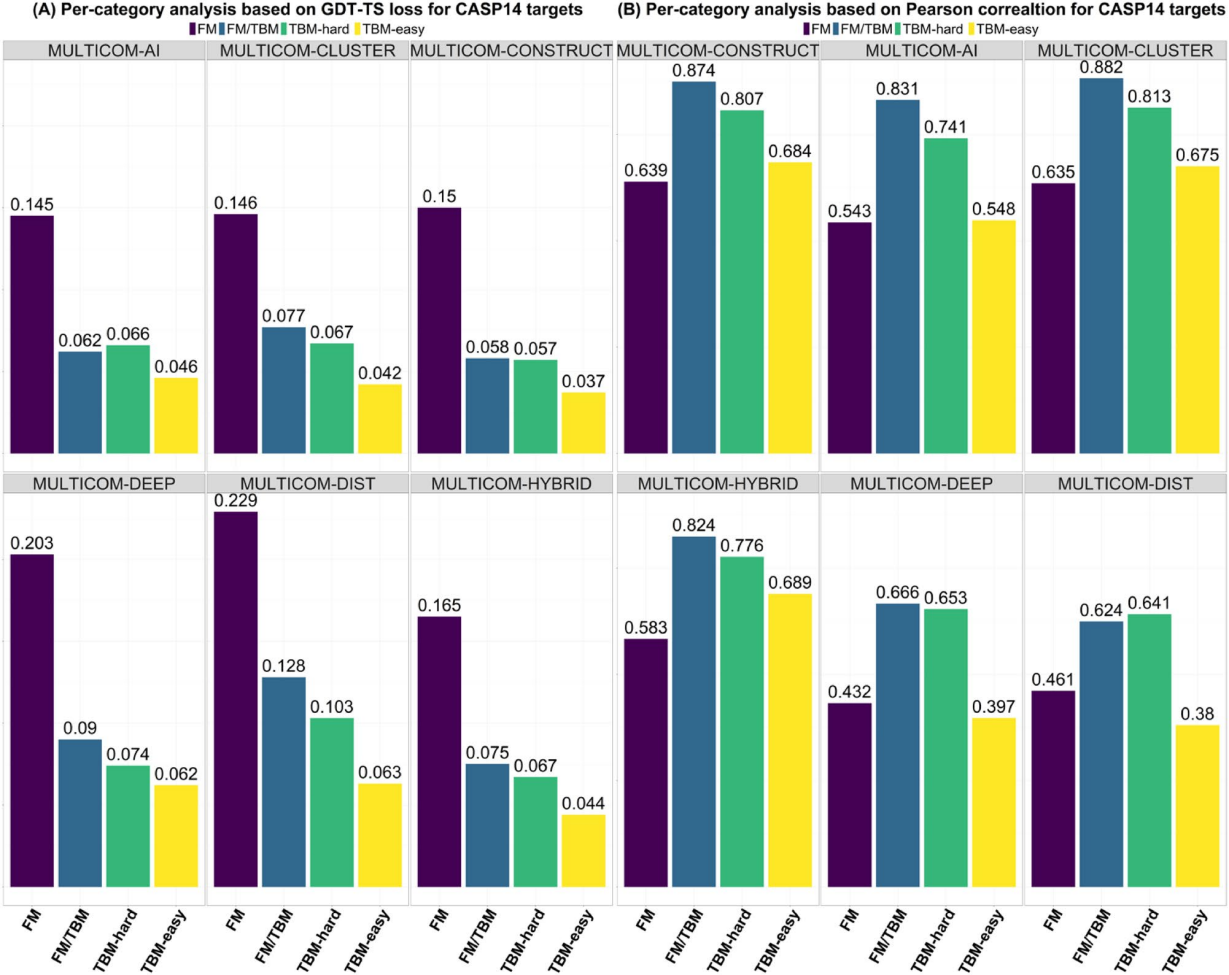
The performance of MULTICOM EMA predictors on four different categories of targets (FM, FM/TBM, TBM-hard, and TBM-easy). (**A**) GDT-TS ranking loss. (**B**) Pearson’s correlation.

Figure 4A illustrates six MULTICOM EMA predictor’s performance in each target category. The multi-model methods consistently outperform the single-model methods in all the categories, indicating that there is still a significant room for single-model methods to improve. For instance, on the TBM-easy targets, four multi-model methods have very close GDT-TS loss (0.044), which is 33.3% lower than the single-model methods’ loss (0.062). MULTICOM-CONSTRUCT obtains the lowest loss (0.057) on TBM-hard targets, while MULTICOM-DIST gets the highest loss (0.103). On the FM/TBM targets, MULTICOM-CONSTRUCT has the lowest loss of 0.058, 34% lower than MULTICOM-DEEP’s 0.09. On the most challenging FM targets, MULTICOM-AI has the lowest loss of 0.145, while MULTICOM-DEEP and MULTICOM-DIST get 0.203 and 0.229 loss, respectively. The results show the GDT-TS loss is lower on easier targets than harder targets for all the MULTICOM EMA predictors generally, indicating that it is still easier to rank the models of easy targets than hard targets.

Figure 4B shows the largely similar trend in Pearson’s correlation evaluation. The four multi-model methods perform better than the two single-model methods. On FM targets, MULTICOM-CONSTRUCT achieves the highest correlation coefficient (0.639), and MULTICOM-CLUSTER gets a very similar correlation score (0.635). MULTICOM-CONSTRUCT’s correlation score is 39% higher than that of MULTICOM-DEEP (0.432). On FM/TMB targets, MULTICOM-CLUSTER result is the best (0.874), 40% higher than MULTICOM-DIST’s score (0.624). MULTICOM-CLUSTER attains 0.813 correlation coefficient on TBM-hard targets and MULTICOM-DIST has lowest correlation coefficient (0.641). On the easiest TBM-easy targets, MULTICOM-CONSTRUCT, MULTICOM-CLUSTER and MULTICOM-HYBRID have the correlation score of around 0.68, while two single-model methods perform worse on these targets. But there is some difference between the evaluation based on the correlation and the GDT-TS ranking loss. The predictors achieve the best performance on FM/TBM targets according to the correlation, but not on the easiest TBM-easy targets according to the ranking loss.

**Figure 5:**
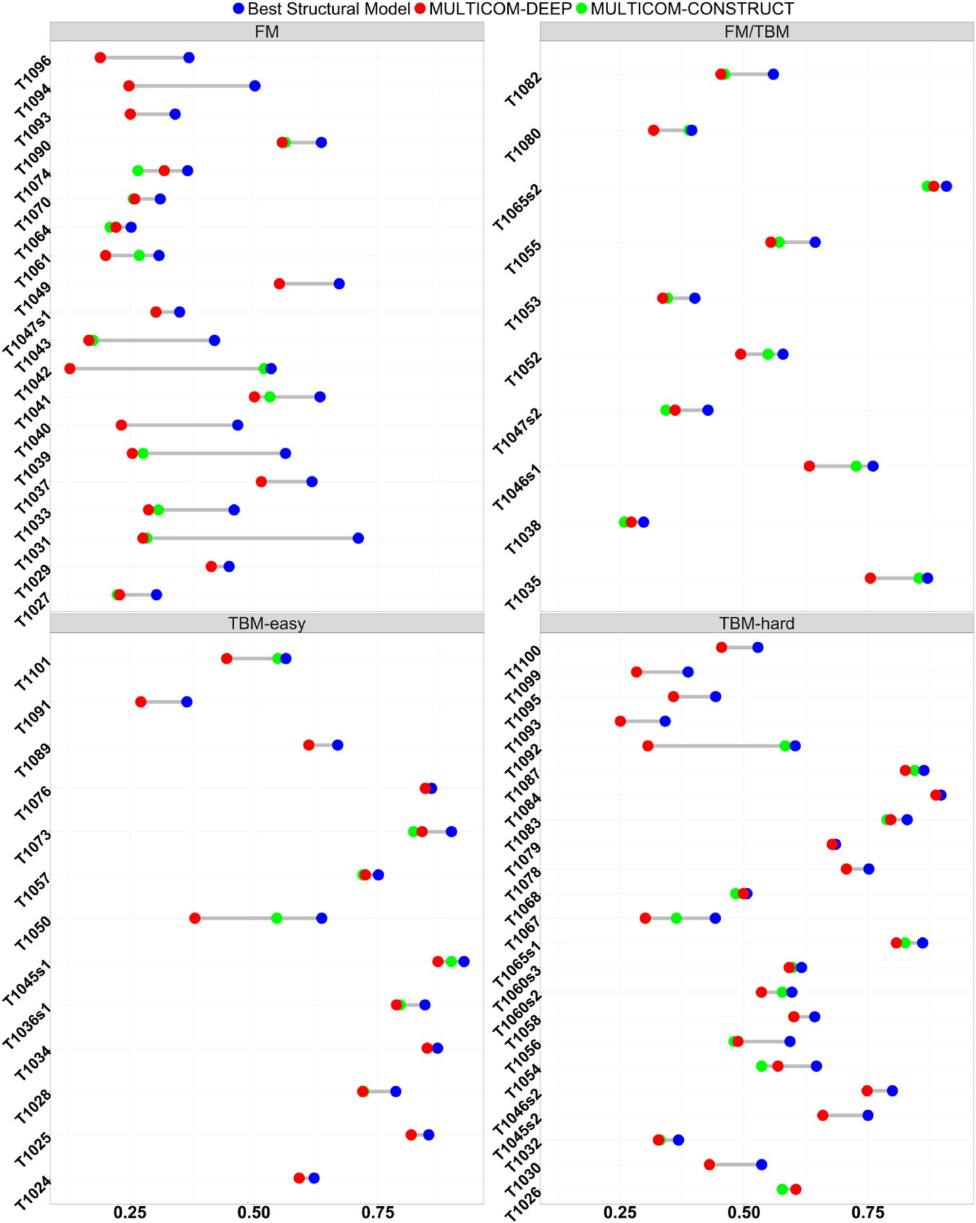
The GDT-TS score of the best structural model (blue dots) for a target, the top model selected by MULTICOM-CONSTRUCT (green dots), and the top model selected by MULTICOM-DEEP (red dots). Closer to a blue dot, lower the loss of the top model represented by a green/blue dot. If a green/blue red dot overlaps with a blue dot, the GDT-TS loss is 0.

Figure 5 is the per-target comparison of the GDT-TS scores of the truly best structural model, the top model selected by a multi-model method—MULTICOM-CONSTRUCT and a single-model method—MULTICOM-DEEP. On most targets (33 targets), the performance of MULTICOM-CONSTRUCT is better than MULTICOM-DEEP. The biggest performance gap occurs on T1042, for which the top model selected by MULTICOM-CONSTRUCT has a true GDT-TS score of 0.5346, four times more than 0.1289 of MULTICOM-DEEP. On 17 targets, MULTICOM-CONSTRUCT and MULTICOM-DEEP have the same performance.

### Comparison with other CASP14 EMA predictors

CASP14 released the whole server’s overall and per-target performance in ranking structural models. Table 3 is the summary of the GDT-TS loss and the Local Distance Difference Test (LDDT)^47^ loss of top 20 out of 68 predictors that predicted the quality of the models of most CASP14 targets. LDDT is a superposition-free score measuring the local distance difference among all atoms between predicted and reference structures. LDDT score reveals the different structural quality of the model compared to the GDT-TS score. One limitation for GDT-TS is that a large domain tends to dominate the global model superposition, while a small domain may not be taken into consideration appropriately in the score calculation. The LDDT score considers the domain movement effect to overcome this limitation. Both metrics have been widely used in CASP to assess EMA methods. According to the results, MULTICOM-CONSTRUCT, MULTICOM-AI, MULTICOM-CLUSTER, MULTICOM-HYBRID is ranked first, second, third, and tenth in terms of GDT-TS loss, respectively. MULTICOM-DEEP was at 20th among all the EMA predictors and 8th among the single-model EMA predictors. That the multi-model EMA methods such as MUTICOM-CONSTRUCT performs better than single-model EMA predictors such as MULTICOM-DEEP is largely because the former integrates single-model quality features (e.g., energy scores and contact/distance-based features) and the pairwise similarity between structural models together. We expect that the performance gap due to the lack of input information could be mitigated by enlarging the dataset used to train single-model EMA predictors as seen in DeepAccNet.

**Table 3:** Top 20 CASP14 EMA Predictors ranked by GDT-TS loss and LDDT Loss, respectively. Here, the original, official GDT-TS loss of each predictor is normalized into the range [0, 1]. Bold font stands for MULTICOM predictors. * and Italic font denote the single-model methods.

#	CASP14 predictors ranked by GDT-TS loss	GDT-TS loss	CASP14 predictors ranked by LDDT loss	LDDT loss
1	**MULTICOM-CONSTRUCT**	0.07356	BAKER-ROSETTASERVER*	4.112
2	**MULTICOM-AI**	0.07924	VoroCNN-GDT*	4.708
3	**MULTICOM-CLUSTER**	0.08023	BAKER-experimental*	4.885
4	MUfoldQA_G	0.08201	tFold-IDT*	5.227
5	MESHI_consensus	0.08404	VoroCNN-GEMME*	5.322
6	BAKER-ROSETTASERVER*	0.08407	**MULTICOM-CONSTRUCT**	5.436
7	BAKER-experimental*	0.08453	VoroCNN*	5.803
8	ModFOLD8*	0.08497	Wallner	5.914
9	Bhattacharya-Server	0.08512	Ornate	6.056
10	**MULTICOM-HYBRID**	0.08606	EMAP_CHAE	6.132
11	Yang_TBM	0.08795	MESHI_consensus	6.147
12	Wallner	0.08931	VoroMQA-dark*	6.241
13	DAVIS-EMAconsensus	0.09009	ProQ3D*	6.279
14	EMAP_CHAE	0.09166	LamoureuxLab	6.453
15	ModFOLDclust2	0.09401	***MULTICOM-DEEP***	6.575
16	VoroCNN-GDT*	0.0969	Bhattacharya-server	6.66
17	GraphQA*	0.0983	**MULTICOM-HYBRID**	6.686
18	Yang-Server*	0.09851	**MULTICOM-AI**	6.748
19	ModFOLD8_rank*	0.10238	tFold-CaT*	6.814
20	MULTICOM-DEEP*	**0.10341**	VoroMQA-stout*	6.834

Different from GDT-TS loss, in terms of LDDT loss, the ranks of MULTICOM predictors are lower. Four MULTICOM predictors are ranked among top 20, and MULTICOM-CONSTRUCT is ranked sixth. The lower ranking of MULTICOM EMA predictors in terms of LDDT loss is partially because they were trained to predict GDT-TS score instead of LDDT score.

### Case study

**Figure 6:**
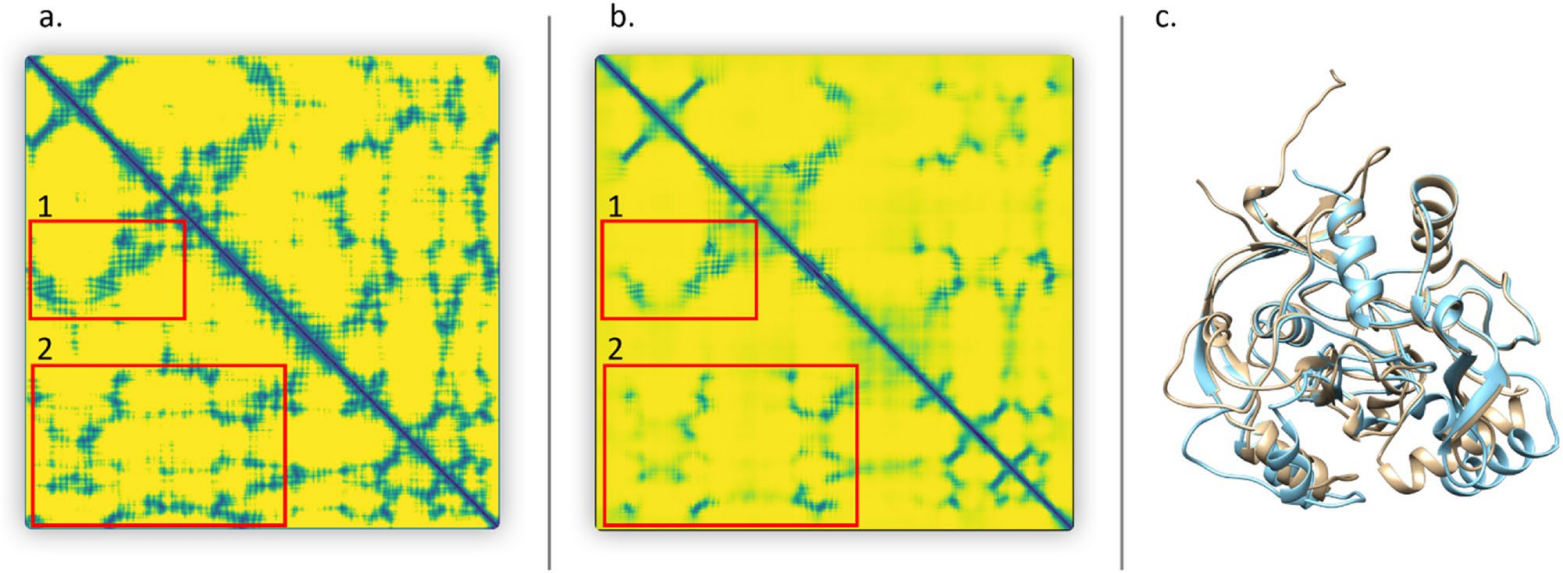
A good EMA example (T1028). (**A**) true distance map (darker collar means shorter distances). (**B**) predicted distance map. (**C**) top model selected by a MULTICOM predictor (MULTICOM-AI) (light blue) versus true structure (light yellow), both protein structures were visualized by Chimera^50^ (version 1.15, https://www.cgl.ucsf.edu/chimera/). The GDT-TS ranking loss is 0. Red rectangles in the maps highlight some long-range contacts.

One successful prediction example made by MULTICOM EMA predictors is shown in Fig. 6. The predicted distance map is similar to the true distance map of the target. Two MULTICOM EMA predictors (i.e., MULTICOM-AI, MULTICOM-CLUSTER) successfully rank the best model at the top, resulting in a loss of 0, and the best model’s GDT-TS score is 0.7861. The precision of top L/2 is 95.21% and top L/5 is 96.55%.

**Figure 7:**
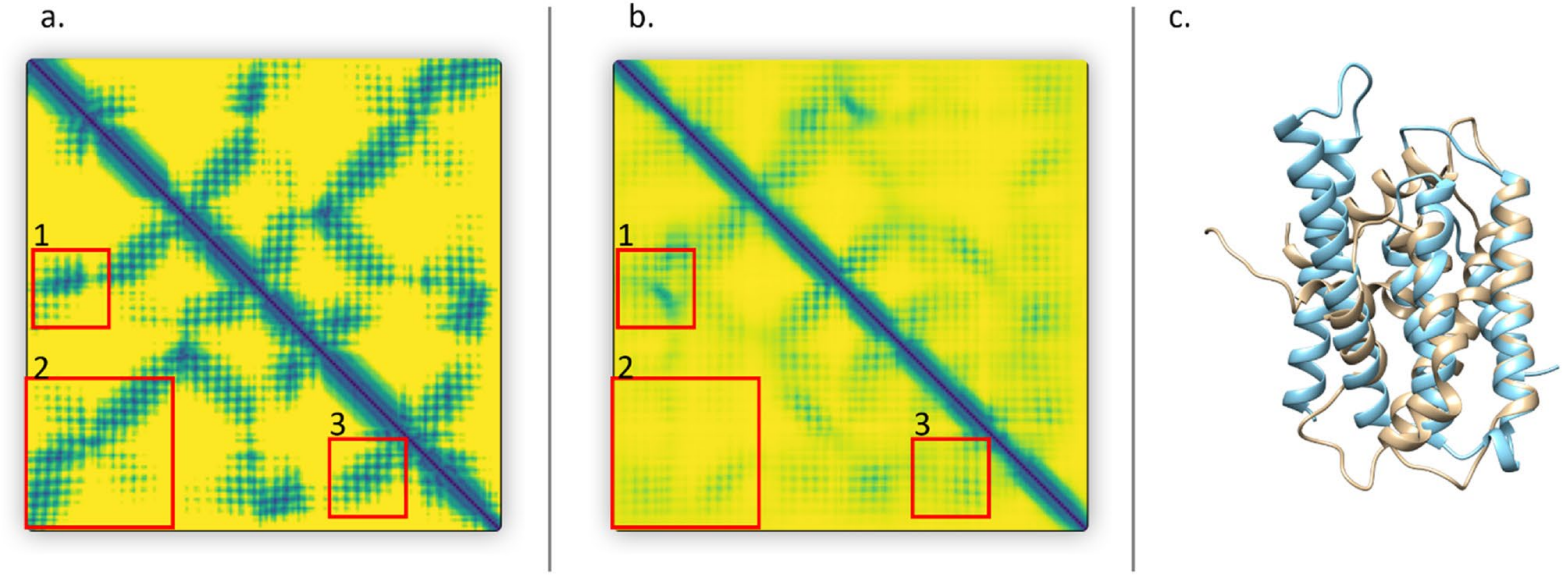
A failed example (T1039). (**a**) the true distance map. (**b**) the predicted distance map. (**c**) top model selected by MULTICOM-AI (light blue) versus true structure (light yellow), both protein structures were visualized by Chimera^50^ (version 1.15, URL: https://www.cgl.ucsf.edu/chimera/). Red boxes highlight regions that the true map and the predicted map differ a lot. The GDT-TS loss of MULTICOM-AI is 0.289.

Figure 7 illustrates a failed example (T1039), on which MULTICOM-AI has a high GDT-TS ranking loss of 0.289 and the best model’s GDT-TS score is 0.5637. The predicted distance map and the true distance map of this target are very different. Particularly, a lot of long-range contacts are not predicted. The precision of top L/2 long-range contacts (L: sequence length) calculated from the predicted distance map is 13.58% and the precision of top L/5 long-range contacts is 18.75%.

## Discussion

The average GDT-TS loss of our best MULTICOM EMA predictors on the CASP14 dataset (e.g., 0.07–0.08) is higher than the average loss (e.g., 0.05) on the CASP13 dataset, suggesting that it is harder to rank the CASP14 models than the CASP13 models. It may be due to the fact most models of CASP13 or even CASP8-12 were generated by the traditional modeling methods, but most models in CASP14 were generated by new distance-based protein structure modeling methods such as trRosetta^44^ developed after CASP13, which may have some different properties from the CASP8-13 models. Therefore, the generalized prediction performance of MULTICOM EMA predictors trained on CASP8-12 models performed worse on CASP14 models than CASP13 models. Consequently, it is important to create larger model datasets produced by new model generation methods to train EMA methods in the future. This may also partially explain why the new distance-based features used in MULTICOM-HYBRID, MULTICOM-DEEP, and MULTICOM-DIST do not seem to improve the performance of EMA over the MULTICOM predictors using only contact-based features on the CASP14 dataset, even though they are mostly accurate enough to build the tertiary structural models according to our tertiary structure prediction experiment in CASP14. Therefore, using the distance information predicted by a distance predictor in model accuracy estimation different from the distance predictors used in tertiary structure prediction is desirable. Besides, further improving the accuracy of distance predictions, particularly for challenging FM targets, can improve the effects of distance-based features on ranking models. Finally, instead of using the expert-curated features derived from distance maps as input, it can be more effective to allow deep learning to automatically learn relevant features for the estimation of model accuracy from raw distance maps or 3D coordinates of structural models.

## Conclusion and future work

We developed several deep learning EMA predictors, blindly tested them in CASP14, and analyzed their performance. Our multi-model EMA predictors performed best in CASP14 in terms of the average GDT-TS loss of ranking protein models. The single-model EMA predictors using inter-residue distance features also delivered a reasonable performance on most targets, indicating the distance information is useful for protein model quality assessment. However, estimating the accuracy of models of some hard targets remains challenging for all the methods. The better ways of using distance features, more accurate distance prediction for hard targets, and larger training datasets generated by the latest protein tertiary structure prediction methods in the field are needed to further improve the performance of estimating model accuracy. Moreover, instead of predicting one kind of quality score (e.g., GDT-TS or LDDT score) for a structural model, it is desirable to predict multiple quality scores via multi-task machine learning to meet the different needs in different situations.
